# Endo-Periodontal Lesions in Endodontically Treated Teeth with Periapical Pathology

**DOI:** 10.3390/diagnostics15131663

**Published:** 2025-06-30

**Authors:** Mihaela Sălceanu, Anca Melian, Cristina Dascălu, Cristian Giuroiu, Corina Concita, Claudiu Topoliceanu, Diana Melian, Andreea Frumuzache, Sorina Mihaela Solomon, Maria-Alexandra Mârţu

**Affiliations:** 1 Department of Odontology-Periodontology and Fixed Restorations, Faculty of Dental Medicine, University of Medicine and Pharmacy “Grigore T. Popa”, 700115 Iasi, Romania; mihaela.salceanu@umfiasi.ro (M.S.); giuroiu.cristian@umfiasi.ro (C.G.); corina-alexandra.concita@umfiasi.ro (C.C.); claudiutopoliceanu@yahoo.com (C.T.); sorina.solomon@umfiasi.ro (S.M.S.); maria-alexandra.martu@umfiasi.ro (M.-A.M.); 2 Discipline of Medical Informatics and Biostatistics, Faculty of Dental Medicine, University of Medicine and Pharmacy “Grigore T. Popa”, 700115 Iasi, Romania; cristina.dascalu@umfiasi.ro; 3 Dental student, Faculty of Dental Medicine, University of Medicine and Pharmacy “Grigore T. Popa”, 700115 Iasi, Romania; meliandiana98@yahoo.com; 4 Endodontics Resident, Faculty of Dental Medicine, University of Medicine and Pharmacy “Grigore T. Popa”, 700115 Iasi, Romania; andreeageorgiana83@yahoo.com

**Keywords:** endo-periodontal lesions, risk, predictor, root canals, periodontal pocket

## Abstract

**Background/Objectives:** The aim of this study was to identify and assess the independent risk factors and potential predictors for endo-periodontal lesions (EPLs) in endodontically treated teeth with periapical pathology. **Methods**: The study group included 90 patients (35 men, 55 women; mean age 47.96 ± 13.495 years) with 126 endodontically treated teeth. Following clinical examinations and radiologic evaluation, 50 patients were diagnosed with endo-periodontal lesions (EPLs) in 64 molars (test group); the control group included 62 endodontically treated teeth without EPLs diagnosed in 40 patients. The independent variables were assessed as risk factors for EPLs. The relationship between patients’ demographic and clinical features and endo-periodontal status was assessed using Chi-squared tests for categorical variables and Student’s *t*- or Mann–Whitney tests for continuous variables, depending on data distribution. The potential risk factors were characterized by calculating Odds Ratios (ORs) with 95% confidence intervals. The variables included in the multivariate logistic regression model were selected based on their clinical relevance and statistical significance in the univariate analysis. To evaluate the combined effect of the identified risk factors, a binary logistic regression model was constructed using the Enter method. **Results:** Out of the 126 endodontically treated molars with periapical pathology, 64 teeth (50.8%) were diagnosed with endo-periodontal lesions (EPLs). Patients aged ≥60 years were significantly more represented in the EPL group (32.8%) compared to the control group (12.9%) (*p* = 0.024). Probing pocket depth ≥ 4 mm was present in 85.9% of teeth with EPLs versus only 30.6% in teeth without EPLs (*p* < 0.001). Probing pocket depth (PPD) ≥ 4 mm was the strongest predictor (OR = 13.830) and remained significant after adjustment in multivariate analysis (OR = 6.585). PPD ≥ 3.625 mm showed a strong association in univariate analysis (OR = 12.587) and preserved significance in the multivariate model (OR = 6.163). **Conclusions:** This study highlights age ≥ 60 years and PPD ≥ 4 mm as the most significant independent risk factors for EPLs, emphasizing the need for early periodontal assessment in endodontically treated teeth with periapical pathology. While PPD greater than 3.625 mm is a strong indicator of the presence of EPLs, other factors such as MBL (marginal bone loss) and occlusal considerations appear to have indirect roles in EPL development in endodontically treated teeth with periapical lesions.

## 1. Introduction

Endo-periodontal lesions (EPLs) are defined as the acute or chronic pathological communication between pulpal and periodontal tissues, which may have originated in the apical periodontium, in the lateral periodontium, or as a combined lesion between the two biological spaces [1]. EPLs are classified according to signs and symptoms impacting their prognosis and treatment. The most recent classification, World Workshop on the Classification of Periodontal and Peri-Implant Disease (2017), emphasizes an individualized approach to diagnosing and treating EPLs, integrating both periodontal and endodontic considerations. According to this classification, EPL categories are as follows [1,2,3]:

- EPLs with Necrotic Pulp—lesions originating from endodontic infection (e.g., pulp necrosis) that extends to the periodontal tissues. These lesions are characterized by the presence of a deep periodontal pocket, purulent exudate, swelling, sensitivity to percussion, and potential fistula formation.
- EPLs with Vital Pulp—lesions develop when periodontal disease affects the apical portion of the root leading to secondary pulpal inflammation, with vital pulp tissue. These lesions are characterized by the presence of a periodontal pocket and clinical signs of periodontal involvement without signs of endodontic infection.
- Combined EPLs—lesions resulting from concurrent endodontic and periodontal in-fections that unite over time. Pulp and periodontal tissues are independently affected, developing simultaneously extensive tissue destruction. Combined EPLs are responsible for more than 50% of general tooth loss [4].

Ruetters et al. (2022) [5] was the first to report an epidemiological study of EPLs prevalence according to the new classification, on 866 patients (18,963 teeth). In that study EPL prevalence was 4.9% (patient-related) and 0.4% (tooth-related).

EPLs are characterized by a bidirectional influence, where infection or inflammation in one tissue affects the other due to anatomical and vascular pathways between the pulp and periodontal ligament, particularly through lateral canals, apical foramina, or dentinal tubules [6,7,8,9]. The microbial flora responsible for EPLs comprise a polymicrobial biofilm dominated by anaerobic bacteria such as *Porphyromonas*, *Prevotella*, *Fusobacterium*, and *Treponema*. These pathogens are known for their virulence factors, facilitating the ability of bacteria to evade host defenses, degrade extracellular matrix components, and induce severe inflammatory responses. The composition and activity of the microbial biofilm vary depending on the pulpal or periodontal primary source of infection [10,11,12,13,14,15].

Research on endo-periodontal lesions using extracted DNA identified over 60 bacterial species. Among these, *E. faecalis*, *P. micra*, *M. timidum*, *F. alocis*, and *F. fastidiosum*, were the most frequent species in root canals, while *P. micra*, *E. faecalis*, *S. constellatus*, *T. forsythia*, *A. actinomycetemcomitans*, *V. parvula*, and *F. alocis* were the most encountered species in periodontal pockets, illustrating the diverse microbial community in lesions [16,17,18,19,20]. Recent research in molecular microbiology of microbial biofilms highlighted the complexity of these infections, supporting personalized therapeutic approaches to eradicate pathogenic bacteria while preserving healthy tissue [21,22,23,24,25].

Predisposing factors for EPLs include iatrogenic causes (root perforations, fractures, improper treatments); traumatic factors (fractures, root resorptions); and systemic pathology (diabetes, osteoporosis, immunosuppression) [26,27]. The etiological complexity of EPLs is challenging in determining patient prognosis and requires multidisciplinary therapy, including periodontal therapy, endodontic therapy, and others, but there is still much debate about the appropriate timing of periodontal therapy and root canal therapy [28,29,30].

Poor prognosis of teeth was reported when grade 2–3 EPLs, treated non-surgically, were associated with risk factors such as increased pocket depth (PD) and clinical attachment loss (CAL), multirooted teeth, smoking, as well as severe generalized periodontitis [31,32,33]. Prognosis of non-surgical therapy of combined EPLs was correlated to the level of the Oral Hygiene Index, the loss of attachment, the clinical crown-to-root ratio, the periapical index, as well as the number of root canals [34]. Also, smoking, age, tooth group, presence of occlusal trauma, improper coronal restorations, presence of caries adjacent to restorations, periapical pathology, and periodontitis were significantly associated with EPLs. The most important prognostic predictors of EPLs were pulp vitality and bone loss [27,35].

Complications of EPLs include odontogenic sinusitis, with EPLs representing the most common odontogenic etiologies (49.5%), followed by apical periodontitis (32.0%) and periodontitis (8.7%) [36]. The clinical success of EPL treatment was significantly improved in recent years due to technological advancements in diagnostic and treatment methods (cone beam computed tomography, endodontic microscope, mineral trioxide aggregates and Biodentine, periodontal regenerative therapies). Combined regenerative endodontic and periodontal therapy can lead to successful outcomes even for teeth with grade 3 EPLs [37,38,39,40]. While therapeutic strategies must disrupt biofilm architecture and combat biofilm-associated diseases [41,42,43], risk assessment of each tooth with EPL must be implemented in patients with iatrogenic complications or advanced periodontitis [44].

There is a paucity of studies investigating independent risk factors for EPLs in molars that have previously undergone root canal treatment and have periapical pathology—a clinically relevant but inadequately investigated situation. By targeting this specific subset of patients, our study offers practical diagnostic insights that are currently limited in the existing literature.

Our study suggests a new clinically significant point of view since the literature about endo-periodontal lesions (EPLs) around endodontically treated molars with periapical pathology is still a debated topic. In contrast to the prior literature, which often investigates endodontic or periodontal risk factors in isolation, our study utilizes an integrated diagnostic approach, documenting independent risk factors via both univariate and multivariate analyses. From a clinical point of view, this study highlights the necessity of early periodontal evaluation and follow-up of endodontically treated teeth with periapical lesions to avoid the development or aggravation of EPLs. The results might guide clinical protocols and risk calculation tools and pave the way for individualized therapeutic strategies. An innovation of this study was differentiating between independent and associative risk factors, aiding clinicians in making evidence-based decisions regarding diagnosis and prognosis.

The aim of this study was to identify and assess the independent risk factors and potential predictors for endo-periodontal lesions (EPLs) in endodontically treated teeth with periapical pathology.

## 2. Materials and Methods

This study adhered to the principles outlined in the Declaration of Helsinki and was approved by the Ethics Department of the University of Medicine and Pharmacy “Grigore T. Popa” (Nr. 334/16 July 2023) in Iași. All patients participating in the study provided informed consent by signing a consent form approved by the ethics committee of “Grigore T. Popa” University of Medicine and Pharmacy in Iași. To determine the necessary sample size for this study power analysis was conducted, utilizing the Periapical Index (PAI) score as the primary outcome variable. A significance level (α) of 0.05 and statistical power of 90% with a large effect size (Cohen’s d = 0.8) were employed. Considering these elements, a total of 68 patients (34 patients per group) were required to achieve sufficient statistical power. The calculation was performed using G*Power analysis (version 3.1).

This was a retrospective study that included a study group of 90 patients (35 men, 55 women; mean age 47.96 ± 13.495 years) with 126 endodontically treated teeth. The patients attended a private dental clinic between the years 2022 and 2024. The inclusion criteria were as follows: (1) patients with periodontitis presenting endo-periodontal lesions; (2) patients who completed treatment for both endodontic and periodontal lesions, including acceptable quality of nonsurgical root canal treatment and periodontal initial therapy; (3) follow-up maintained over six months; (4) availability of records with all clinical parameters and radiographic examination results; and (5) patients older than 18 years. The exclusion criteria were as follows: (1) patients who had root fracture or cracking, root canal, or pulp chamber perforation or external root resorption; (2) patients who underwent periodontal or periapical surgery; (3) patients who had systemic diseases, such as hypertension, diabetes, heart disease, liver disease, or kidney disease; or (4) patients with incomplete medical records.

All patients were evaluated by one calibrated experienced clinician (M.S.). Diagnosis of endo-periodontal pathology was performed by using major and minor diagnostic criteria (Abott and Salgado). Major diagnostic criteria were as follows: (1) periodontal probing depth (PD) ≥4 mm, (2) clinical attachment loss (CAL) ≥3 mm, and (3) patients with pulp symptoms, such as spontaneous pain history or negative or altered pulp vitality tests. Minor diagnostic criteria were (1) red and swollen gums, (2) bleeding on probing, (3) varying degrees of alveolar bone resorption in radiological images, (4) occlusal discomfort, (5) tooth mobility (TM), (6) discoloration associated with pulp necrosis, (7) purulent exudate, (8) sinus tract [Abott and Salgado]. The new classification (2018) of the American Academy of Periodontology (AAP) for endo-periodontal lesions is as follows: grade 1—narrow deep pocket in one tooth surface, grade 2—wide deep pocket in one tooth surface, and grade 3—deep pocket in more than one tooth surface in patients with periodontitis [1,26].

The independent variables assessed as risk factors for EPLs were as follows: dental groups, location (maxillary/mandibular), coronal restoration type (amalgam/composite fillings, full coverage crown), presence of intracanal posts, quality of the root canal fillings, quality of the coronal restoration marginal sealing, recurrent caries, periodontal status (periodontal grade, PP≥4 mm), and the presence of antagonist teeth.

**Figure 1:**
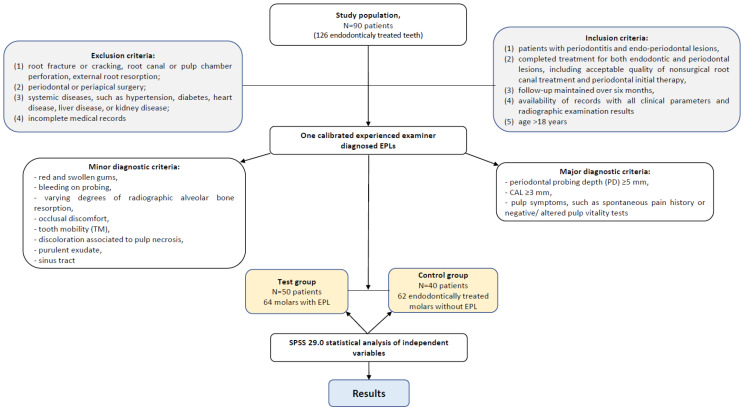
Flowchart detailing selection of patients.

The presence of periodontal pathology was defined according to the classification proposed in 2017 in the World Workshop on the Classification of Periodontal and Peri-Implant Disease [1,2,3], where periodontitis of the endodontically treated tooth was clinically diagnosed by the presence of bleeding on probing (BOP) and probing pocket depth (PPD) ≥4 mm (Figure 1).

The Orstavik Periapical Index (PAI) [45] was used to assess periapical status in the study groups. In multi-rooted teeth, the worst PAI score was considered. Failure of endodontic therapy was defined by the clinical signs and symptoms of apical inflammation, widening of the periodontal ligament, or the presence of any periapical radiolucency with PAI > 2 [46].

The status of the root canal fillings and coronal restoration marginal sealing were assessed as ‘adequate’ or ‘poor’ using the radiographic criteria shown in Table 1 [46,47].

### Statistical Analysis

The statistical analyses were performed in SPSS 29.0. Qualitative variables were described by frequency distributions, while quantitative variables were characterized by descriptive statistics (mean and standard deviation). The relationship between patients’ demographic and clinical features and endo-periodontal status was assessed using Chi-squared tests for categorical variables and Student’s *t*- or Mann–Whitney tests for continuous variables, depending on data distribution. The potential risk factors were characterized by calculating Odds Ratios (OR) with 95% confidence intervals. To evaluate the combined effect of the identified risk factors, a binary logistic regression model was constructed using the Enter method. The variables included in the multivariate logistic regression model were selected based on their clinical relevance and statistical significance in the univariate analysis (*p* < 0.05): age ≥ 60 years; presence of opposing tooth; PPD ≥ 4 mm; periodontal status stage II, III, or IV; PPD cut-off ≥ 3.625 mm; MBL-D ≥ 2.250 mm; and MBL-M ≥ 2.750 mm. All variables were entered simultaneously into the multivariate model. The model’s adequacy was evaluated using the Hosmer–Lemeshow goodness-of-fit test (*p* = 0.378). The model improved the accuracy in identifying the endo-periodontal status from 50.8% to 76.2%, explaining 46.6% of the variation in the dependent variable (according to the Nagelkerke R-squared coefficient); its sensitivity was 82.8% and its specificity was 69.4%, indicating that the model was appropriately constructed and the identified predictors were relevant. For the quantitative variables used to characterize the periodontal status, ROC analysis was performed in order to identify the cut-off values specific for teeth with endo-periodontal lesions. The quantitative variables relevant to evaluate the periodontal status were PPD, probing pocket depth; MBL (marginal bone loss), D (distal); MBL (marginal bone loss), M (mesial). A p-value < 0.05 was considered statistically significant, while *p* < 0.01 was evaluated as statistically highly significant.

## 3. Results

Following clinical examinations and radiologic examination, 50 patients (mean age 50.72 ± 13.820 years) were diagnosed with endo-periodontal lesions (EPLs) in 64 molars (test group); the control group included 62 endodontically treated teeth without EPLs diagnosed in 40 patients (mean age 44.50 ± 12.393 year) (Table 2).

**Figure 2:**
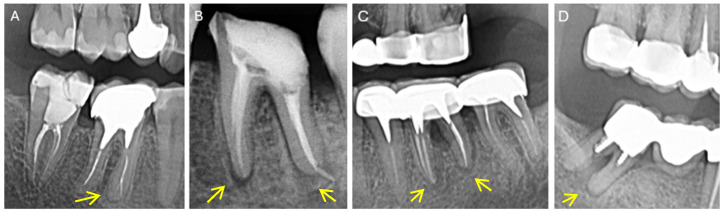
Endo-periodontal lesions (without root damage) in endodontically treated teeth with periapical lesions: (**A**) Grade 1 (4.6.–4.7) (**B**) Grade 2 (3.6) (**C**) Grade 3 (3.6.) (**D**) Grade 3 (4.7) (EPL classification according to Herrera et al., 2018 [26]).

The radiological aspects of combined endo-periodontal lesions (classified according to [26]) are revealed in Figure 2A–D.

The distribution of the endodontically treated teeth in the test group (*n* = 64) and control group (*n* = 62) according to socio-demographic and clinical parameters is revealed in Table 3. Significant statistical differences were found between the test and control groups for the parameters age group (*p* = 0.024) and the presence of opposing tooth (*p* = 0.008). Most EPLs in the test group were found in the teeth of the age group 40–59 years (45.3%), followed by the age group ≥60 years (32.8%) and age group 20–39 years (21.9%). The presence of PPD ≥4 mm was found in 85.9% teeth with EPLs (periodontitis patients), while only 30.6% of teeth without EPLs had PPD ≥4 mm. PPD < 4 mm were found in only 14.1% of teeth with EPLs (non-periodontitis patients), while 69.4% of teeth without EPLs were found with PPD < 4 mm. The presence of PPD ≥ 4 mm (*p* < 0.001 **) was associated with highly significant differences between the test (EPLs teeth) and control groups (teeth without EPLs). The absence of significant statistical differences between the test and control groups was recorded for gender (*p* = 0.671), smoking status (*p* = 0.263), OHI (Oral Hygiene Index) (*p* = 0.126), location (*p* = 0.425), coronal restoration type (direct composite restoration, *p* = 0.885; full coverage crown, *p* = 0.885; intracanal posts, *p* = 0.713), coronal restoration quality (*p* = 0.794), root canal filling quality (*p* = 0.680), recurrent caries (*p* = 0.862), and periodontal status (*p* = 0.104) (Table 3).

The distribution of endo-periodontal lesions according to severity classification [26] was as follows: 26.57% EPL grade 1; 34.37% EPL grade 2; 39.06% EPL grade 3 (Table 4).

Clinical (PPD) and radiological parameters (MBL) between the test and control groups are compared in Table 5. Teeth with EPLs exhibit significantly deeper periodontal pockets (4.70 ± 1.044 mm) compared to those without EPLs (3.81 ± 0.789 mm) (*p* < 0.001). Teeth with EPLs show greater distal bone loss (3.28 ± 1.545 mm) than teeth without EPLs (2.19 ± 0.884 mm), indicating more severe alveolar resorption (*p* < 0.001). The presence of EPLs is associated with substantially increased mesial bone loss (4.04 ± 1.85 mm) compared to teeth without EPLs (2.73 ± 1.13 mm) (*p* < 0.001).

**Figure 3:**
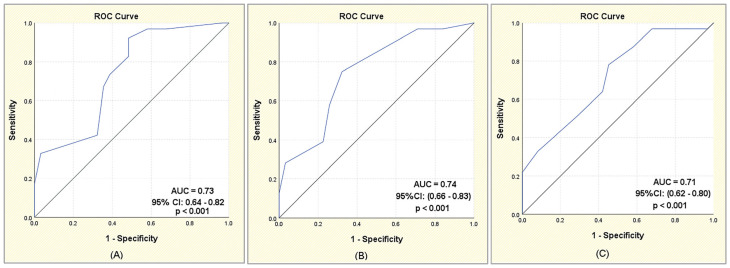
ROC curves for PPD (**A**), MBL-D (**B**), and MBL-M (**C**) values.

Table 6 and Figure 3A–C show Receiver Operating Characteristic (ROC) analysis for PPD, MBL-D, and MBL-M values. Cut-off values for EPLs in endodontically treated teeth with periapical lesions were 3.62 mm for PPD, 2.25 mm for MBL-D, and 2.75 mm for MBL-M. A probing pocket depth (PPD) greater than 3.62 mm is a strong indicator of the presence of EPLs, with a high sensitivity of 92.2%. Distal marginal bone loss (MBL-D) over 2.25 mm has a sensitivity of 75% and a specificity of 67.7%, making it a relatively reliable parameter in identifying EPLs. Mesial marginal bone loss (MBL-M) over 2.75 mm has moderate predictive capacity, with a sensitivity of 78.1% but lower specificity (54.8%). All three parameters (PPD, MBL-D, and MBL-M) have AUC values above 0.7, indicating moderate accuracy in detecting EPLs.

The results of logistic regression analysis are shown in Table 7. Age ≥ 60 years was significantly associated with EPLs in both univariate (OR = 3.297, *p* = 0.008) and multivariate analysis (OR = 5.393, *p* = 0.014), indicating that older individuals have a higher risk of developing EPLs. The presence of an opposing tooth showed a significant association in univariate analysis (OR = 3.333, *p* = 0.008), but after adjustment in the multivariate model, it was no longer significant (OR = 1.688, *p* = 0.372), suggesting that while occlusal forces might play a role, they are not independent predictors. PPD ≥ 4 mm showed the strongest statistical association with the presence of EPLs in univariate analysis (OR = 13.83, *p* < 0.001) and remained a significant independent variable in the multivariate model (OR = 6.58, *p* = 0.012).

However, it is important to note that logistic regression analysis does not establish a causal relationship but rather indicates a strong correlation between the observed parameters. The cut-off value of PPD ≥ 3.62 mm showed a strong association in univariate analysis (OR = 12.58, *p* < 0.001) but had borderline statistical significance in multivariate analysis (OR = 6.16, *p* = 0.053), suggesting that deep periodontal pockets are relevant but may be influenced by other factors. Patients’ periodontal stage (II, III, or IV) (vs. stage I) was associated with EPLs in univariate analysis (OR = 2.50, *p* = 0.041), but its significance was lost in the multivariate model (OR = 0.424, *p* = 0.22). The same phenomenon was noticed in the case of marginal bone loss (MBL-D ≥ 2.25 mm), highly significant in univariate analysis (OR = 6.300, *p* < 0.001) and marginal bone loss (MBL-M ≥ 2.75 mm), with strong association in univariate analysis (OR = 4.33, *p* < 0.001)—all these parameters lose their significance after adjustment (OR = 0.45, *p* = 0.238).

## 4. Discussion

Endo-periodontal lesions (EPLs) have also been referred to as retrograde periodontitis in the past, and since it affects the dental pulp as well as the periodontium, these structures communicate with one another through pathological communication pathways [36,48].

Our study sought to evaluate the influence of clinical parameters such as age, probing pocket depth (PPD), marginal bone loss (MBL), periodontal status, and occlusal factors. By differentiating between significant independent risk factors and those with indirect associations, this research aims to enhance diagnostic accuracy and guide clinical decision-making for the management of EPLs. In our study, endodontically treated teeth with periapical lesions and EPLs exhibited significantly worse periodontal parameters (PD, MBL) compared to those without EPLs. The statistically significant p-values across all parameters (*p* < 0.001) reinforce the strong association between EPLs and periodontal disease.

The significant association between age ≥ 60 years and EPLs in endodontically treated teeth with periapical lesions aligns with previous studies highlighting age-related changes in periodontal and periapical tissues. Older patients often exhibit compromised healing potential, increased periodontal attachment loss, and cumulative exposure to risk factors such as periodontal disease, which may explain their higher susceptibility to EPLs [49,50].

A PPD ≥ 4 mm was observed in 85.9% of teeth diagnosed with EPLs (in patients with periodontitis), whereas only 30.6% of teeth without EPLs exhibited PPD ≥ 4 mm. The presence of PPD ≥ 4 mm demonstrated a highly significant association (*p* < 0.001) between the test group (teeth with EPLs) and the control group (teeth without EPLs), indicating a strong correlation between increased periodontal pocket depth and the development of EPLs. In our study, PPD greater than 3.62 mm was strongly associated with the presence of EPLs, showing very high sensitivity (92.2%). Deep periodontal pockets contain complex anaerobic microbiota, including several highly virulent species such as *Porphyromonas gingivalis*, *Tannerella forsythia*, *Treponema denticola*, *Fusobacterium nucleatum*, and *Parvimonas micra* [17,18,19,20,21]. The bidirectional route of infection supports the concept of a PPD > 4 mm as an independent predictor of EPL development in endodontically treated teeth [6,42,50,51]. A distal marginal bone loss greater than 2.25 mm was associated with EPLs. For this parameter, sensitivity and specificity were more balanced compared to PPD, making it a reasonably reliable predictor of EPL presence in endodontically treated teeth with periapical lesions. A mesial marginal bone loss greater than 2.75 mm is also a predictor of EPLs, with moderate sensitivity (78.1%) but lower specificity (54.8%). These results suggest that the cut-off values for PPD, MBL-D, and MBL-M can be used as predictive factors in the diagnosis of EPLs. However, they should be combined with other clinical criteria to improve diagnostic accuracy. The significant correlation between increased PPD and the presence of EPLs underscores the critical role of periodontal health in endodontic outcomes [51]. Higher PPD indicates more extensive periodontal destruction and higher values of the periodontal pockets that may facilitate the entry of bacterial products and inflammatory mediators into the apical or lateral canal region of the root canal system. Such anatomical connections between the periodontal ligament and pulp tissue (lateral canals, apical foramina, or accessory canals) can allow for bidirectional spread of infection. Deep periodontal pockets may synergistically fuel the pathogenesis of EPLs via anatomical and microbial mechanisms [6,42,51]. Also, a research group concluded that BOP and PPD > 3.5 mm were significant risk factors for endodontic failure [52]. A research group reported the association between periapical lesions, severe periodontitis, and furcation involvement [44]. However, in our study, periodontitis in stages II-IV was significantly associated with EPLs only in univariate analysis.

The presence of bone loss was a predictor of EPLs in our study. Although distal MBL and mesial MBL were strongly associated with EPLs in univariate analysis, they lost significance in the multivariate model. This finding suggests that bone loss is an important clinical indicator of disease severity, reflecting the cumulative effects of periodontal disease and periapical inflammation. Shiggaon et al. (2024) reported a strong association between bone loss and EPLs and concluded that bone loss is a predictor of EPLs [35].

In our study, the OHI (*p* = 0.12) was not significantly different between test and control groups, indicating that oral hygiene was not a major risk factor for EPLs. Location of the affected teeth (*p* = 0.42) does not show a significant difference, meaning EPLs are not more prevalent in a specific dental arch or region.

In our study, the presence of an opposing tooth was significantly associated with EPLs in univariate analysis, a result confirmed by Shiggaon et al. (2024) [35]. This result was explained by the role of the occlusal forces in the progression of periodontal disease in already compromised teeth [53].

Despite its detrimental impact on periodontal tissue [54,55,56,57,58], in our study smoking (*p* = 0.26) did not influence the presence of EPLs in endodontically treated teeth. Smoking was considered by some research groups a significant prognostic factor for developing apical periodontitis [59], while others have indicated that smoking is not significantly associated with apical periodontitis when adjusting for factors such as the quality of root canal fillings [60,61,62].

Coronal restoration type (direct composite restoration, *p* = 0.88; full coverage crown, *p* = 0.88; intracanal posts, *p* = 0.71), coronal restoration quality (*p* = 0.79), root canal filling quality (*p* = 0.68), and recurrent caries (*p* = 0.86) did not influence significantly the onset of EPLs in endodontically treated teeth with periapical lesions. This result was surprising considering that a poorly treated root canal can facilitate the movement of endodontic pathogens toward neighboring periodontal tissues [6,42,50], while defective restorations can allow the penetration of bacteria and toxins at the interface of the restoration and the tooth, leading to recurrent dental caries [57,58] and subsequent periapical infection [52,63,64]. Further studies involving larger sample sizes are requested to validate these findings and better understand the influence of both coronal and endodontic restorative factors on the onset and development of EPLs.

Our study identified PPD ≥ 4 mm and age ≥ 60 years as independent risk factors for EPLs and noted that early periodontal evaluation and risk assessment in endodontically treated teeth are important. Also, the proposed clinical cut-off values for PPD and MBL may help clinicians in recognizing high-risk teeth due to the tissue destruction preceding disease activity.

The distinction between independent risk factors and associated risk factors not only facilitates a more accurate diagnostic process but can also aid treatment planning, especially with regard to the timing of concurrent periodontal and endodontic therapies. Such findings might potentially enhance long-term outcomes and tooth retention by initiating preventive treatment in high-risk groups, particularly in the elderly with early signs of periodontitis.

Considering the bidirectional influence of periodontal and periapical pathology risk factors, patient-associated factors [65,66,67,68] are involved both in the pathogenesis of periodontitis and endo-periodontal lesions, while iatrogenic errors during endodontic treatment can also influence the prognosis of affected teeth [41,68,69]. Other causal/risk factors include the clinical experience and skills of the endodontist or generalist dentist [44]. All these factors must also be investigated in further studies.

One of the strengths of this study is the assessment of risk factors for endo-periodontal lesions (EPLs) in molars previously subjected to endodontics and currently showing periapical pathology. The statistical analysis is performed stringently based on univariate and multivariate models adjusting for independent risk factors. Also, we suggested clinical cut-off values for probing pocket depth (PPD) and marginal bone loss (MBL), which could be useful as a reference for clinical practice. Another merit is the separation between independent and side risk factors, so it adds up to a better diagnosis. All assessments were performed by a well-calibrated and experienced examiner, which therefore increases the consistency and reliability of the collected data.

The combination of deep PPD and altered periapical healing creates optimum environment for the interaction of periodontal and endodontic infections. These common mechanisms of pathogenesis suggest that an integrated diagnostic and therapeutic approach is warranted for the management of EPLs [70,71,72,73,74].

Future research should explore the interplay between periodontal, endodontic, and systemic factors to improve predictive models and clinical management strategies, including possible adjuvant treatment options in compromised patients such as those with diabetes or cardiovascular or renal disease or in immunocompromised individuals [75,76].

An interesting aspect is the relationship between EPLs and oral cancer. There are several studies detailing the link between periodontal pathology and oral cancer; however, the literature on EPLs and oral cancer is extremely limited and involves mostly clinical cases [36,77,78,79]. Another potential future direction is the evaluation of which factors could lead to the extraction of endo-periodontally affected teeth that lead to extraction and subsequent implant therapy in order to devise clear clinical protocols that clinicians could follow for maximum clinical success [80,81,82,83,84].

In the future, biomarkers associated with tissue inflammation, bone resorption, and periodontal destruction may offer additional insight into disease activity and prognosis, especially in systemic conditions like diabetes or osteoporosis. However, their clinical application remains limited due to cost, variability, and lack of standardization.

Limitations of the study include a relatively small sample size, limited paraclinical investigations and lack of microbiological testing. The lack of long-term follow-up limits the speculation on EPL progression or treatment response. The lack of microbiological or immunological testing also hinders any deeper understanding of the mechanisms behind the lesions’ pathogenesis. Moreover, as a single-center study, there is a possibility of certain bias on patient selection or local peculiarities of the medical act.

## 5. Conclusions

This study highlights age ≥ 60 years and PPD ≥ 4 mm as the most significant independent risk factors for EPLs, emphasizing the need for early periodontal assessment in endodontically treated teeth with periapical pathology. While PPD greater than 3.625 mm is a strong indicator of the presence of EPLs, other factors such as MBL (marginal bone loss) and occlusal considerations appear to have indirect roles in EPL development in endodontically treated teeth with periapical lesions.

Univariate and multivariate analysis indicated that periodontitis severity alone does not independently predict EPLs in endodontically treated teeth with periapical lesions.

## Figures and Tables

**Table 1:** Parameters of root canal fillings [46,47].

Parameters	Criteria	Description
Parameters of root canal filling quality		
Density of root canal filling	Adequate	Absence of gaps in the root filling or between root filling and root canal walls
	Poor	Presence of gaps in the root filling or between root filling and root canal walls
Length of root canal filling	Adequate	Root filling ending ≤ 2 mm from radiographic apex
	Poor	Root filling over the radiographic apex or root filling > 2 mm from radiographic apex
Conicity of root canal filling	Adequate	Continuously tapering funneled root canal preparation from the canal entrance to the apex, and cross-sectional diameter narrower at every point apically
	Poor	Inconsistent taper from the canal entrance to the apical part of the filling, or root filling deviated from the original canal
Parameters of coronal restoration quality		
	Adequate	Any permanent coronal restoration with intact radiographic aspect
	Poor	Any permanent coronal restoration with detectable signs of recurrent caries or open margins or presence of temporary coronal restoration
	Missing	Absence of permanent or temporary coronal restoration

*p* < 0.05 statistically significant.

**Table 2:** Socio-demographic and clinical parameters of studied population patients (patients with EPLs vs. teeth without EPLs).

Parameter	Absent E-P (Control)		E-P (Test)		Total		*p*-Value
	*n*	%	*n*	%	*n*	%	
Global	40	100.0	50	100.0	90	100.0	
Gender							0.809
M	15	37.5	20	40.0	35	38.9	
F	25	62.5	30	60.0	55	61.1	
Age (years) (mean, SD)	Globally: 44.50 ± 12.393		Globally: 50.72 ± 13.820		Globally: 47.96 ± 13.495		0.029 *
	M: 45.13 ± 13.309		M: 48.70 ± 14.357		M: 47.17 ± 13.832		
	F: 44.12 ± 12.077		F: 52.07 ± 13.820		F: 48.45 ± 13.380		
	*p* = 0.806		*p* = 0.404		*p* = 0.663		
Age group (years)							0.280
20–39	13	32.5	12	24.0	25	27.8	
40–59	23	57.5	27	54.0	50	55.6	
≥60	4	10.0	11	22.0	15	16.7	

* *p* < 0.05 statistically significant.

**Table 3:** Socio-demographic and clinical parameters of test and control groups.

	Absent EPLs (Control)		EPLs (Test)		Total		*p*-Value
Parameter	*n*	%	*n*	%	*n*	%	
Global	62	100.0	64	100.0	126	100.0	
Gender							0.67
M	21	33.9	24	37.5	45	35.7	
F	41	66.1	40	62.5	81	64.3	
Age group (years)							0.02 *
20–39	21	33.9	14	21.9	35	27.8	
40–59	33	53.2	29	45.3	62	49.2	
≥60	8	12.9	21	32.8	29	23.0	
Smoking	19	30.6	14	21.9	33	26.2	0.26
OHI							0.12
1	6	9.7	2	3.1	8	6.3	
2	33	53.2	44	68.8	77	61.1	
3	23	37.1	18	28.1	41	32.5	
Location (MX/MD)							0.42
MX	20	32.3	25	39.1	45	35.7	
MD	42	67.7	39	60.9	81	64.3	
Coronal restoration type							
Composite resin filing	25	40.3	25	39.1	50	39.7	0.88
Class I	6	24.0	6	24.0	12	24.0	1.00
Class II	19	76.0	19	76.0	38	76.0	
Full-coverage crown	37	59.7	39	60.9	76	60.3	0.88
Intracanal posts	11	17.7	13	20.3	24	19.0	0.71
Coronal restoration quality							0.79
Adequate	5	8.1	6	9.4	11	8.7	
Poor	57	91.9	58	90.6	115	91.3	
Root canal filling quality							0.68
Adequate	2	3.2	4	6.3	6	4.8	
Poor—reasons:	60	96.8	60	93.8	120	95.2	
Under-filling	53	85.5	47	73.4	100	79.4	0.09
Over-filling	6	9.7	7	10.9	13	10.3	0.81
Inadequate homogeneity	55	88.7	54	84.4	109	86.5	0.47
Inadequate taper (conicity)	58	93.5	57	89.1	115	91.3	0.37
Opposing tooth							0.008 **
YES	42	67.7	56	87.5	98	77.8	
NO	20	32.3	8	12.5	28	22.2	
Recurrent caries							0.86
YES	53	85.5	54	84.4	107	84.9	
NO	9	14.5	10	15.6	19	15.1	
PPD ≥ 4 mm							<0.001 **
YES	19	30.6	55	85.9	74	58.7	
NO	43	69.4	9	14.1	52	41.3	
Periodontal status (stage)							0.10
I	18	29.0	9	14.1	27	21.4	
II	27	43.5	26	40.6	53	42.1	
III	13	21.0	23	35.9	36	28.6	
IV	4	6.5	6	9.4	10	7.9	
Periodontal status (grade)							0.014 *
A	22	35.5	6	9.4	28	22.2	
B	24	38.7	36	56.3	60	47.6	
C	16	25.8	22	34.4	38	30.2	

EPLs—endo-periodontal lesions; OHI—Oral Hygiene Index; MX—maxillary; MD—mandibular; PPD—probing pocket depth. * *p* < 0.05 statistically significant; ** *p* < 0.001 highly statistically significant.

**Table 4:** Endo-periodontal lesions classification [26].

	*n*	%
Grade 1	17	26.57%
Grade 2	22	34.37%
Grade 3	25	39.06%

**Table 5:** Comparison of clinical and radiological parameters between test and control groups.

	Absent E-P (Control)	E-P (Test)	Total	*p*-Value
PPD (mean, SD)	3.81 ± 0.78	4.70 ± 1.04	4.26 ± 1.02	<0.001 **
MBL-D (mean, SD)	2.19 ± 0.88	3.28 ± 1.54	2.75 ± 1.37	<0.001 **
MBL-M (mean, SD)	2.73 ± 1.13	4.04 ± 1.85	3.40 ± 1.66	<0.001 **

** *p* < 0.01 statistically highly significant. PPD—probing pocket depth; MBL—marginal bone loss; D—distal; M—mesial.

**Table 6:** ROC analysis for PD, MBL-D, MBL-M indices.

	AUC (95% CI)	*p*	Cut-off Value	Sensitivity	Specificity
PPD	0.73 (0.64 ÷ 0.82)	0.000 **	3.62	0.92	0.51
MBL-D	0.74 (0.66 ÷ 0.83)	0.000 **	2.25	0.75	0.67
MBL-M	0.71 (0.62 ÷ 0.80)	0.000 **	2.75	0.78	0.54

** *p* < 0.01 statistically highly significant. PPD—probing pocket depth; MBL—marginal bone loss; D—distal; M—mesial.

**Table 7:** Univariate and multivariate logistic regression for the association of study variables with endo-periodontal status (OR in relation to the assessed parameters).

	Univariate Analysis		Multivariate Analysis	
	OR (95% CI)	*p*-Value	OR (95% CI)	*p*-Value
Age ≥ 60 years	3.29 (1.33 ÷ 8.16)	0.008 **	5.39 (1.398 ÷ 20.79)	0.014 *
Opposing tooth	3.33 (1.33 ÷ 8.30)	0.008 **	1.68 (0.535 ÷ 5.32)	0.372
PPD ≥ 4 mm	13.83 (5.69 ÷ 33.60)	<0.001 **	6.58 (1.511 ÷ 28.70)	0.012 *
Periodontal status II, III, or IV	2.50 (1.02 ÷ 6.10)	0.041 *	0.42 (0.107 ÷ 1.67)	0.221
Cut-off PPD ≥ 3.625	12.58 (4.44 ÷ 35.60)	<0.001 **	6.16 (0.974 ÷ 38.99)	0.053 *
MBL-D ≥ 2.250	6.30 (2.89 ÷ 13.70)	<0.001 **	1.39 (0.504 ÷ 3.87)	0.520
MBL-M ≥ 2.750	4.33 (1.99 ÷ 9.41)	<0.001 **	0.45 (0.121 ÷ 1.69)	0.238

* *p* < 0.05 statistically significant; ** *p* < 0.01 statistically highly significant.

## Data Availability

Data supporting reported results can be provided by the corresponding authors upon reasonable request.
